# What prevents midwifery quality care in Bangladesh? A focus group enquiry with midwifery students

**DOI:** 10.1186/s12913-018-3447-5

**Published:** 2018-08-15

**Authors:** Malin Bogren, Kerstin Erlandsson, Members of the Midwifery Faculty Master’s degree holders in Sexual and Reproductive Health and Rights, Hasne Ara Akter, Zohra Khatoon, Sumona Chakma, Krishna Chakma, Anawara Anowara, Hasina Akter, Swarnalata Sarker, Montaj Mohol, Rehana Kathun, Rahima Khatun, Nilima Rani Bormon, Jhama Rani Mondal, Yamin Ara Khatun, Nurmohol Khatum, Shirin Akhter, Momtaz Bergum, Fazila Khatum, Mamotaz Begum, Shamsunnahar Bergum, Farida Yesmin, Arse Ara Begum, Halima Akter, Dalia Akter, Rehana Akter, Sufia Begum, Renoara Akter, Syeada Yesmin, Merry Chowdhury, Lucky Das, Ulrika Byrskog

**Affiliations:** School of Education, Health and Social Studies, Dalarna University, 791 88 Falun, Sweden

## Abstract

**Background:**

With professional midwives being introduced in Bangladesh in 2013, the aim of this study was to describe midwifery students perceptions on midwives’ realities in Bangladesh, based on their own experiences.

**Method:**

Data were collected through 14 focus group discussions that included a total of 67 third-year diploma midwifery students at public nursing institutes/colleges in different parts of Bangladesh. Data were analyzed deductively using an analytical framework identifying social, professional and economical barriers to the provision of quality care by midwifery personnel.

**Results:**

The *social barriers* preventing midwifery quality care falls outside the parameters of Bangladeshi cultural norms that have been shaped by beliefs associated with religion, society, and gender norms. This puts midwives in a vulnerable position due to cultural prejudice. *Professional barriers* include heavy workloads with a shortage of staff who were not utilized to their full capacity within the health system. The reason for this was a lack of recognition in the medical hierarchy, leaving midwives with low levels of autonomy. *Economical barriers* were reflected by lack of supplies and hospital beds, midwives earning only low and/or irregular salaries, a lack of opportunities for recreation, and personal insecurity related to lack of housing and transportation.

**Conclusion:**

Without adequate support for midwives, to strengthen their self-confidence through education and through continuous professional and economic development, little can be achieved in terms of improving quality care of women during the period around early and late pregnancy including childbirth.The findings can be used for discussions aimed to mobilize a midwifery workforce across the continuum of care to deliver quality reproductive health care services. No matter how much adequate support is provided to midwives, to strengthen their self-confidence through education, continuous professional and economic development, addressing the social barriers is a prerequisite for provision of quality care.

**Electronic supplementary material:**

The online version of this article (10.1186/s12913-018-3447-5) contains supplementary material, which is available to authorized users.

## Background

Addressing quality of care is central in reducing maternal and newborn morbidity and mortality and achieving the Sustainable Development Goals’ (SDG) health-related targets for women and newborns [1]. The period around childbirth is the most critical for saving the maximum number of lives. Hence, ensuring provision of quality care in this area is a serious concern for many health systems [2].

In low-income countries, such as Bangladesh where resources are scarce, the provision of quality care around the time of childbirth is especially challenging [3], as it requires equitable access to educated, skilled, and motivated health professionals [4, 5] working in a functioning health system [3, 6]. With respect to the global priority where midwives are an important element of the health workforce, and seen as fundamental to the provision of quality care for women in early and late pregnancy and around childbirth [2, 7–9], the Government of Bangladesh has scaled up the availability of educated midwives into its health system [10].

However, there remains an enormous shortage of midwives in Bangladesh and elsewhere [11]. Research indicates that midwives who are part of the workforce often experience disrespect, subordination by medical professionals, and gender discrimination at work and in the community [12]. There is also evidence of the urgent need to address these barriers experienced by midwives, as well as gender inequality and rights issues that underline this [13]. Consequently, until these barriers are targeted, efforts to strengthen quality midwifery care will have little effect [12].

As midwifery as a profession in its own right is new to Bangladesh, with the first diploma level midwives becoming licensed as recently as 2016 [10], there have not yet been any studies carried out to assess barriers preventing midwives from providing quality midwifery care in Bangladesh. Similarly, no studies demonstrating midwives’ realities in the workplace and/or in the community/country have been conducted. Bangladesh is aiming to become a middle-income country by 2021 [14] and the targets in place to end preventable maternal and newborn deaths are dependent on improving quality midwifery care [3, 15]. With the recent introduction of midwives to the Bangladeshi health system [16], the increasing rate of caesarian births [17, 18], and the country’s increasing maternal mortality and newborn ratio [19], the perspectives of future midwives placed in the intersection between theory and practice are central for the identification of social, economic, and professional barriers that prevent quality midwifery care in the country**.** Hence, the aim of this study was to describe midwifery students perceptions on midwives’ realities in Bangladesh, based on their own experiences.

## Methods

### Design

A focus group enquiry was conducted at 14 public nursing institutes/colleges out of the total of 38 in Bangladesh. The conceptual framework: Barriers to the provision of quality care by midwifery personnel [13] was guiding the data collection and analysis of data. A total of 67 third-year midwifery students in Bangladesh participated. In this paper, good quality care is aligned with the International Confederation of Midwives Core Competencies [20] and viewed from the perspective of a wider reproductive health service system [8]. Informed verbal and written consent were obtained from the participants before the focus group discussions started. Ethical clearance was obtained from Directorate General Nursing and Midwifery, on February 21, 2017.

### Setting and participants

Bangladesh is a lower middle-income country [21] where poverty and income inequalities is a persistent challenge [22]. Despite interventions as provision of family planning and education of girls, gender inequalities impact on the socio-economic development, particular of women from the rural areas [23]. With approximately 3.1 million live births each year [24], 47 % of deliveries take place in a health facility, which contributes to the overall low ratio of skilled attendance at birth of 50% and a high maternal mortality ratio (MMR) of approximately 196 maternal deaths for every 100,000 live births [19].

Since its inception, the midwifery diploma programme has been embedded in nursing institutes/colleges in Bangladesh, and is currently being offered at 38 public institutes/colleges, which are geographically distributed across the country [10]. The focus group discussions took place at 14 of these public nursing institutes/colleges. A nursing college is a government institution affiliated with a tertiary-level hospital with a high level of referrals from primary level, whilst nursing institutes are situated at district level, and affiliated with general hospitals where midwifery students are placed for their practical learning placements. The yearly intake of midwifery students, all female, is 25–50 places at each institution. The educational institutions are residential, and students come from both rural and urban areas.

Sixty-seven third-year diploma midwifery students were purposively [25] selected to participate in the focus group discussions, based on their willingness and availability. The age of the participants ranged from 19 years to 23 years, all were female, unmarried without own children.

### Material

The conceptual framework of Filby et al. [13] describes barriers connected to the context of childbirth and related to midwifery as being organized into three categories; social, economic, and professional. This framework has become recognized in national, regional, and international dialogues on quality midwifery care provided by midwives. Our understanding of this conceptual framework is that the social barriers to the provision of quality of care can be seen as underlying links to the socially and culturally constructed context of childbirth as well as gender inequality. The economic barriers in the framework are understood as low or absent wages, informal payments and a lack of governmental financial commitment, and professional barriers are understood as lack of investment in quality midwifery education; weak or absent regulation; inadequate numbers of staff; lack of affordable transport; weak facility management and poor working conditions.

Based on this framework, a semi- structured topic guide was developed for this study (see Additional file 1) with open-ended questions, such as, *What are the midwives’ realities in Bangladesh from a social/economic/professional perspective? What are the existing barriers to providing quality midwifery care in Bangladesh?*

### Data collection

Data were collected through 14 focus group discussions at 14 public nursing institutes/colleges in different parts of Bangladesh. The criteria was to select institutes/colleges throughout the different regions of Bangladesh and to include institutes/colleges in both urban and semi-urban settings.

Faculty members at the different institutions/colleges conducted the focus group discussions in pairs using the semi structured topic guide, and each group included 4–7 participants. The faculty members, similar the data collectors, were undertaking a master degree education in sexual and reproductive health, in which qualitative data collection methods were included, theoretically and practically. All focus group discussions took place in a separate room at the participants’ institute/college. Participants were informed orally and in writing about the aim of the study and that they were free to withdraw participation at any time, without any consequences. They were encouraged to talk freely, and were asked follow-up questions when necessary. Each focus group discussion was conducted in English or Bangla when English was not understood. They were all digitally recorded, and lasted between 30 and 60 min. Any parts conducted in Bangla were thereafter translated into English by an experienced translator. The translator who was not a member of the focus group discussions, had access to the topic guide and was retrospectively listening to all recordings made during the group discussions in order to detect any translation errors during the discussions. Thereafter, all focus group discussions were transcribed verbatim.

### Analysis

The transcripts were analyzed using qualitative deductive content analysis inspired by Elo and Kyngäs [26]. The manifest analysis was performed in several steps. The first step aimed to gain a sense of the transcripts by answering the question: *What are the social, economic, and professional barriers preventing quality midwifery care to mothers and newborns in Bangladesh,* which involved all the authors reading the transcripts several times. In the second step, meaning units corresponding to social, economic, and professional barriers preventing quality midwifery care were identified. Thirdly, after the meaning units were compared for similar content, they were labeled with codes. Fourthly, as the analysis progressed with the continuous comparison of the codes for similarities and differences, the codes were clustered into emerging sub-categories such as “Being in a vulnerable situation facing cultural prejudice”, “Having a low level of autonomy in a medical hierarchy*”,* and *“*Low and irregular salary and stipends”. The codes were thereafter sorted into the broad pre-set categories related to social, economic, and professional barriers preventing quality midwifery care to mothers and newborns in Bangladesh. After the authors discussed and refined the analysis, a final consensus was reached.

## Results

### Social barriers

The following subcategories reflect the social barriers preventing midwifery quality of care: “It does not start in the workplace but in society”, and “Being in a vulnerable situation facing cultural prejudice”.

#### It does not start in the workplace but in the society

The midwifery students stressed that women in general are neglected and disrespected in Bangladeshi society. This neglect and disrespect of girls and women prevents quality midwifery care. Thus, it does not start in the workplace, but in society itself. This neglect and disrespect starts already when a baby girl is born.

*We witness mothers delivering baby girls and see how the rest of the family members are unhappy. We have also seen the joy expressed when a baby boy is born into the family. Family members have distributed sweets among hospital staff after the birth of a baby boy, however, if a baby girl is born no sweets are distributed. Our society thinks that women cannot do anything* (FGD 6).

The midwifery students described that inequalities began with the families treating girls and boys differently. There is a preference to educate boys, while educational opportunities are often withheld from girls. Parents often wanted their daughters to get married at an early age. In families where this happens, the safety of the girl is a key motivator.

*“When I was given the chance to study at the Chittagong Nursing College my parents were not happy and asked why I needed to study. I asked them why they were asking this question. In reply, my father said ‘Being a women you don’t need higher education’, he feared that “outside” the home a girl is not safe, he also said ‘You will get married soon and look after your household’. I had arguments with them and decided to enroll on the programme anyway, and then when I got this midwifery job my parents were happy”* (FGD 2).

#### Being in a vulnerable situation facing cultural prejudice

According to the midwifery students, Bangladesh’s religion and culture do not permit girls and women to be outside the home at night, but being a midwife requires working night duty. The midwifery students described their parents’ concern because of lack of proper security measures in the workplace. Some midwife students described fear of harassment when working nights. This meant *if a midwife works night shifts she is considered immoral and she has to subject herself to different forms of harassment such as eve teasing – the making of unwanted sexual remarks or advances by a man to a woman in a public place –, and physical and sexual violence* (FGD 2). This reduces the quality of midwifery care provided to mothers giving birth at night.

The midwifery students described how husbands or mothers-in-law were often in charge of the household finances and the decision for a woman to utilize midwifery care or not. This prevented many women from receiving quality midwifery care, as they instead choose to give birth at home. Another barrier to quality midwifery care was cultural beliefs. The midwifery students expressed that there was a lack of acceptance for midwives’ advice about nutrition and rest, and that these were considered contradictory to having a normal birth.

*One pregnant woman was not getting antenatal checkups because her mother-in-law did not permit her. She thought that if the woman goes to a midwife for advice, they would advise her to rest and to eat enough food. The mother-in-law’s belief was that this would lead to a big baby and she would have to suffer through delivering a baby so big that a normal delivery would be impossible, meaning she might need a cesarean section* (FGD 2).

Rumours of midwifery care are widely spread in Bangladesh. The midwifery students talked about how people think that midwives are not competent in providing quality midwifery care, and therefore they do not utilize midwifery services. Another perception by the midwifery students’ was that people think midwives are young and unskilled in supporting a woman through childbirth and maternal and newborn care. The midwifery students wished people knew more about what the midwifery educational programme includes and what midwives do.

*They are not aware that midwives have a diploma level education (FDG 1).*

### Professional barriers

The following subcategories reflect the professional barriers preventing midwifery quality of care: “Lack of professional recognition, heavy workload, and staff shortages”, and “Having a low level of autonomy in a medical hierarchy”.

#### Lack of professional recognition

The midwifery students expressed their concerns about only having limited insights into the national level policy dialogue. The students linked the lack of midwifery representatives in policy making to a number of negative consequences related to quality of care, such as inadequate management, shortage of skilled personnel, and ignorance about the role of midwives in society.

The Bangladesh Government has recently developed a midwifery curriculum and legislative documents to ensure quality midwifery care to mothers and newborns. Some midwifery students responded that midwives were still unaware of their professional rights and limitations. One student gave a concrete example, “*We have a protocol of service but we do not know how many drugs we can prescribe. We do not know how to use the drugs in an emergency situation when a woman is giving birth”* (FGD 2).

The midwifery students perceived that they were not given opportunities to practice their theoretical or clinical knowledge and skills. This was expressed by one midwifery student: *Without proper skills, we will not be able to perform quality midwifery care* (FDG 3). They described that these possibilities were restricted due to several factors. Partly they linked it to weak coordination with health care facilities, where there was competition between nurses, nursing students, intern doctors, and midwifery students when supporting a woman giving birth. Furthermore, there was a gap between their theoretical knowledge and practical experience and there were no clinical teachers.

#### Heavy workload and shortage of staff

In Bangladesh, the number of midwives is limited. The midwifery students described that the government is struggling with deployment of midwives and a shortage of midwives is a barrier to quality care. The shortage of midwives could lead to increased work stress, misunderstandings, and insufficient care.

*“I can’t care for all the women at the same time. When this becomes a problem, the women get angry with us as they don’t understand. On the other hand, when this scenario occurs in the hospital, patients don’t get proper care. Because we are in a hurry to provide treatment to a large number of women, sometimes we mess up. We are not able to manage our workload properly” (FGD 3).*

At village and sub-center levels, there is no 24-h midwifery care due to there being a shortage of midwives. The midwifery students described that the shortage of midwives combined with the large number of women needing support meant that they were unable to meet demand. This led to the midwives becoming exhausted.

#### Having a low level of autonomy in a medical hierarchy

Medical intern doctors dominated midwives’ practice. Senior doctors supported intern doctors. The midwifery students’ perception was that the intern doctors believed that they knew better than the midwives did.

*“We often see intern doctors doing the wrong thing. If we try to tell them that his or her technique is not right, they do not agree. They do not want to know about our skills. On the other hand, they claim that they know better than us” * (FGD 3).

Midwives need to make decisions regarding further action and provision of quality care based on a woman’s condition. However, midwives’ independent decision making power is restricted as is their permission to speak out when they witness malpractice. One midwifery student stated:

*“If we discuss normal delivery and the positive side of it, they (the intern doctors) do not agree with us and say we are wrong. We want to contribute but we aren’t permitted to. Often they (the intern doctors) conduct normal births using the wrong technique. They want to deliver the baby quick and fast. In this scenario, it is evident that the midwives’ skills do not mean anything at all to the doctors*” (FGD 3).

The midwifery students faced a range of restrictions everywhere. They described the competition between the medical professions and how they felt vulnerable and lacked power.

*“One day my colleague and I were putting on gloves and preparing ourselves to help a woman to deliver when suddenly the intern doctor came into the delivery room, shouted at us, and asked us why we were wearing gloves. Why didn’t we call her? We had no power at all over the doctor and had to take our gloves off. Many of us faced this kind of situation on our course (the midwifery education programme)”* (FGD 13).

### Economic barriers

The following subcategories reflect the economic barriers preventing midwifery quality care: Lack of supplies and hospital beds, Low and/or irregular salaries and stipends, Personal insecurity related to lack of housing and transportation, and Lack of security related to lack of housing and transportation, and Lack of recreation.

#### Lack of supplies and hospital beds

Lack of supplies and hospital beds prevent quality midwifery care. When an emergency patient comes to the hospital, lifesaving medication is often unavailable and her relatives need to buy it from the pharmacy. If an emergency happens at night when the pharmacy is closed, the midwives cannot take necessary action to save the woman’s life. One midwifery student described:

*“If an emergency patient comes to the hospital and we are running out of medicine and equipment, we have to ask the patient’s family to buy those things from the pharmacy. It takes more time to provide emergency services to the patient, which places the woman in danger”* (FDG 5).

Women and their relatives had accused midwives of stealing medication, when the fact was that they were out of stock. This was described as: “*Often medicines are out of stock so the women are not getting their medication when they should. This makes the women and relatives get angry and accuse us of stealing medicine. In these cases, the women do not trust us”* (FDG 10).

Autoclave sterilizers were also in short supply. This resulted in midwives having no other choice than to use the same instruments on different women without sterilizing them.

*“I was assisting an intern doctor. A woman gave birth. I cut the umbilical cord and turned around to get the utensils and saw they had been taken away to be used for other woman”* (FDG 11). Situations like this affect hygiene routines.

#### Low and/or irregular salaries and stipends

Salary has a large impact on daily life and low or irregular salaries are a major barrier to quality midwifery care in Bangladesh. A midwife’s salary is low compared to other health care professionals. Their low salaries force midwives to take up part time jobs so it is possible to pay for the education of their own children. “*Working two different jobs makes a midwife exhausted and increases the chances of her not performing appropriately*” (FDG 7).

It was stated that if midwives received salaries equal to the men’s, it would help midwives to maintain a stable economic situation for themselves and their families. This would lead to less stress in midwives’ daily working life. The midwifery students thought equal pay would act as an incentive, and make midwives more eager to provide quality care.

Irregular payments were another economic barrier, especially when midwives did not get their salaries in the first week of the month. Irregular salaries make a midwives’ life stressful which leads to her being unable to perform satisfactorily in her work as a midwife. This prevents quality midwifery care.

Since most midwifery students belong to lower or middle class families, they are dependent on stipends. They are worried about student life and “*Student midwives get stipends, but they are irregular. We have not received any stipends for five months now”* (FGD 6).

#### Insecurity due to lack of housing and transportation

Midwives working in rural areas are not able to perform adequately without safe transportation and housing facilities. Unsafe accommodation is a reality for many midwives. Despite this limitation, midwives are committed to providing quality midwifery care. The midwifery students emphasized their fear of living far away from the hospital as many had bad experiences of eve teasing.

*“One of the sisters told me her story. She works for Upazila Health Complex, and her house is quite far from her workplace. Every day she has to walk there and she has experienced eve teasing by men on the way to work, and it makes her feel unsafe”* (FGD 2).

Another student said: *“Transportation is difficult as a midwife needs to spend extra money, making it a burden on her economy as well as the fact she has to endure physical and sexual violence when travelling by public transport”* (FDG 10).

If midwives are to provide quality midwifery care, they also need support in the form of adequate transportation to the work place, and safe housing when being posted far away. This was a problem linked to difficulties in recruiting midwives. In some cases, midwives had to live in low quality accommodation far from the health facility they worked in for very little income. Living far away from work involves large time and financial costs for midwives which prevents quality midwifery care.

*“Transportation is not always available or affordable. A midwife needs to walk to reach her working place, and this is not possible all the time”* (FDG 2).

Students described that they experienced midwifery being a new discipline as a reason for their being at a disadvantages when it comes to housing.

*“Nursing is a well-established course, it started 100 years ago, so nursing students have their own ego, and they don’t usually mix with us. They have separate floors for living and they do not allow us to go to their rooms. The new candidates for the midwifery courses are forced to live 13 to 14 girls together in a room whereas the nurses only have 10 in a room. We should have a separate building for midwifery students” (FGD 6).*

#### Lack of recreation

Women are responsible for raising children and maintaining their professional work. The lack of time and opportunity for recreation has repercussions on the midwives’ working life. Their possibilities for rest and recreation were limited. Midwifery students described how juggling home life and professional work made them tired and exhausted.

*“Because of the heavy workload, midwives can’t spend time with their husbands and children, which creates family tension”* (FGD10).

They further described that midwives were often not in a position to take enough leave. Lack of recreation prevents quality of midwifery care. “*Working so many hours makes a midwife feel boring and exhausted, which affects the midwifery care.”* (FDG2). “*There is a lack of midwives at their service place so they are not getting regular leave.”* (FGD 5).

## Discussion

This paper presents the perceptions of future midwives on midwives’ realities in Bangladesh of the social, economic, and professional barriers preventing midwives from providing quality midwifery care. This interview study has identified significant barriers across the health system in Bangladesh, despite its attempt to provide quality care during the period around early and late pregnancy and childbirth. Identifying these barriers can help set priorities that can feed into a sustainable health system offering equitable access to health services, with access to personnel, governance and leadership, procurement and distribution of equipment and medicines, and with a financing system and systems for generating and using health information [3, 6, 27]. We will therefore view the findings from this study in the light of a well-functioning health system [27] as the basis to discuss our findings, and suggest practical solutions to improve the provision of quality midwifery care.

Our findings point to that the provision of quality midwifery care falls outside the parameters of cultural norms shaped by beliefs associated with religion, society, and gender norms. This puts midwives in a vulnerable position where they face cultural prejudice. The social barriers preventing quality midwifery care recognized in this study were found to start already when a baby girl is born. This is not unique to Bangladesh but also within the region [28–30]. As such, women’s low status and limited decision-making autonomy contributes towards gender inequality and has a negative impact on the maternal mortality rate due to a lack of access to and utilization of health care [31–33]. At the same time, gender inequality can be seen as a failure in health service delivery. The unpredictable health seeking behavior of women in society does not necessarily imply that care provision is perceived to be of poor quality. Rather, midwives such as those found in this study, balance available resources with feelings of being powerless as women and in their profession, resulting in being overburdened when providing care. This is supported by other studies describing the complexity of barriers affecting midwives [13, 34]. Clearly, any strategy that aims to strengthen the delivery of health services must consider rights-based approaches to gender equality, and thus better reflect women’s status in society with access to and utilization of midwifery care.

In addition to this, the present study found that professional barriers preventing midwifery quality care were reflected by a lack of professional recognition paired with heavy workloads and staff shortages. Interestingly, similar findings are presented in a related South Asian country [35]. Our findings put forward the concept that midwives in Bangladesh are not utilized to their full capacity within the health system. The reason for this is a lack of recognition in the medical hierarchy, leading to midwives having low levels of autonomy. This is in agreement with other research that shows that midwives in the workforce are often treated with disrespect, subordination from medical professionals, and gender discrimination in the workplace [12]. Equally, this seems to be a global challenge, thus the Global Strategy for Human Resources for Health 2030 has stressed the importance of optimizing the performance, quality, and impact of the health workforce [36]. There is an urgent need to mobilize a midwifery health workforce across the continuum of care to deliver quality reproductive health services where and when required. However, based on our findings, and supported by other research, health systems can be strengthened first through a political commitment – through strong policies and leadership [6, 34] with plans to support recruitment, deployment, and retention of autonomously practicing midwives that enable them to deliver quality midwifery care [16, 37]. Although the government of Bangladesh has taken actions in deploying licensed midwives as per international standards into the health system, guided by a code of ethics, job description, career development pathway and with an academic progression for midwives [10], this study shows that there are still gaps in the provision of midwifery quality care. This implies that available policies do not necessarily lead to the provision of midwifery quality care unless financing and support for midwives is comprehensive.

In the present study, economical barriers preventing midwifery quality care were reflected in the lack of supplies and hospital beds, midwives’ low and irregular salary and stipends, lack of opportunities for recreation, and personal insecurity related to lack of housing and transportation; whereof many are similar to those economic barriers previously reported in the framework by Filby et al. [13]. Hence, when resonating against health financing issues, Bangladesh has faced difficulties to set the necessary conditions for achieving effective accountability related to financing and support to midwives. In agreement with Sharma et al. [3], it is therefore suggested that increasing funding for salaries and supplies would positively impact on midwifery quality care during the period around early and late pregnancy including childbirth. Health financing issues faced by many low-income countries are related to the lack of financial resources for health and development overall, and are not specific to quality midwifery care [38, 39]. The findings from this study point to health financing being a key policy instrument to improve midwifery quality care in Bangladesh, and that health care inequalities could be reduced by removing financial barriers to access and preventing the financial hardship expressed by the participants in this study. However, without the right policies and financial allocation systems in place, there is a tendency for inequalities to increase in terms of access to and the provision of quality care and services [18, 38, 40]. Thus, our findings can be considered a starting point for discussions and can be used as evidence to prioritize equitably costing for the provision of quality midwifery care.

### Methodological considerations

The main limitation of this study was that the interviewers in their profession were faculty and the participants were students. Hence, status and power distribution was unequal. If the scenario had have been more equal, the findings may have disclosed even more barriers. Another limitation was the high number of focus group discussion moderators, but to achieve confirmability [25] all focus group discussions followed the same topic guide and structure. Performing focus group discussions in Bangla and in English can be seen as both a strength and a limitation. This could be a strength as the moderators translated the topic guide into Bangla when needed so the students could use Bangla if they did not feel comfortable expressing themselves in English. But it could also be a limitation due to the possible loss of content when translating the interviews into English. Despite these limitations, this study is unique as it is following an international recognized framework [13] contextualized into the Bangladesh setting. Although our intention was not to test the feasibility of the framework, this study design can be transferred to any healthcare professionals and other cultural and social contexts to deepen understanding and identifying strategies for improvements of quality care.

## Conclusion

Without adequate support for midwives, to strengthen their self-confidence through education and through continuous professional and economic development, little can be achieved in terms of improving quality care of women during the period around early and late pregnancy including childbirth. Our findings point to the need to consider strategies to support women leadership, proper midwifery workforce planning, education and training, and effective management of professional midwifery jurisdiction. Despite the impact gender inequality has on the provision on quality midwifery care, the health system needs to continue its tireless support in integrating autonomously practicing midwives into the health system. Thus, our findings can be considered a starting point for discussions aiming to mobilize a midwifery health workforce across the continuum of care to deliver quality reproductive health services, and be used as evidence to prioritize equitably costing for the provision of quality midwifery care.

Finally, no matter how much adequate support is provided to midwives, to strengthen their self-confidence through education, continuous professional and economic development, addressing the social barriers is a prerequisite for provision of quality care.

## Additional file

Additional file 1: Topic guide for focus group discussions on: What prevents quality midwifery care in Bangladesh?. (DOCX 36 kb)

## Abbreviations

FGD: Focus group discussion
ICM: International Confederation of Midwives
MMR: Maternal mortality ratio
SDG: Sustainable Development Goals’
