# Supplementary material for: What prevents midwifery quality care in Bangladesh? A focus group enquiry with midwifery students
Source: BMC Health Serv Res. 2018 Aug 15;18:639. doi: 10.1186/s12913-018-3447-5 (PMC6094463; doi:10.1186/s12913-018-3447-5)
Supplement: Supplementary file 1 — Topic guide for focus group discussions on: What prevents quality midwifery care in Bangladesh?. (DOCX 36 kb) [file 12913_2018_3447_MOESM1_ESM.docx]

**Topic guide for focus group discussions on: What prevents quality midwifery care in Bangladesh?**

You are invited to participate in a focus group discussion that will investigate what prevents quality midwifery care in the Bangladeshi settings. Inspired by the analytical framework; “What prevents quality midwifery care from social, economic and professional perspectives”, this study aims to describe midwifery students perceptions on midwives’ realities in Bangladesh, based on their own experiences.

Important information to all before the discussion starts:

This focus group discussion will discuss the themes from the analytical framework “What prevents quality midwifery care from social, professional and economic perspectives” and will guide the discussion. During the discussion, all participants are free to share their opinions and should be encouraged to speak up. Different opinions are welcome and respect for each ones contributions and opinions are central. All participants are informed that participation is voluntary, it is possible to refrain or withdraw from participation at any time without giving a reason. The data is collected under confidentiality. This means that data will be depersonalized in any publication and stored safely. Handling of the data will be restricted to those directly involved in compiling the publication. This also means that all participants are requested not to spread what has been said in the discussion to anyone outside the group. All participants will be aware, before the discussion starts that the discussion will be digitally recorded. Informed consents are to be filled in by all participants before starting the discussion.

1. ***What are the midwives realities in Bangladesh from a social perspective?***
2. *Free discussion*
3. *If required follow up questions will relate to:*
4. Gender equality
5. Respect
6. Safety and security
7. Empowerment

*Use the following probes when encouraging the participants to talk and discuss:*

- Please describe!
- Does anyone have own experience or experiences you have heard of, that you want to share regarding …..
- Can you please describe how?
- Can you explain why you think this happens in the Bangladeshi setting?
- Do you agree/disagree?
- Can anyone else add something?

1. ***What are the existing barriers to providing quality midwifery care in Bangladesh from a social perspective?***
   - Apart from what we already have discussed, can you describe or discuss any other barriers from a social perspective, which prevents quality midwifery care, as you see it?

*Let everyone tell their feeling and after that:*

Please, describe and discuss why you feel in this way

*If negative feelings, probe:*

Please, discuss what actions could be done which could change these feelings and by whom

1. ***What are the midwives realities in Bangladesh from an economic perspective?***
2. *Free discussion*
3. *If required follow up questions will relate to:*
4. Salary
5. Housing
6. Transportation
7. Leave

*Use the following probes when encouraging the participants to talk and discuss:*

- Please describe!
- Does anyone have own experience or experiences you have heard of, that you want to share regarding …
- Can you explain why you think this happens in the Bangladeshi setting?
- Can you please describe how?
- do you agree/disagree?
- Can anyone else add something?

1. ***What are the existing barriers to providing quality midwifery care in Bangladesh from an economic perspective?***
   - Apart from what we already have discussed, can you describe or discuss any other barrier from an economic perspective, which prevents quality midwifery care, as you see it?

*Let everyone tell their feeling and after that:*

Please, describe and discuss why you feel in this way

*If negative feelings, probe:*

Please, discuss what actions could be done which could change these feelings and by whom

1. ***What are the midwives realities in Bangladesh from a professional perspective?***
2. *Free discussion*
3. *If required follow up questions will relate to:*
4. Policy dialogue
5. Decision making
6. Skills
7. Practice
8. Supplies
9. Equipment
10. Training
11. Staffing
12. Competence
13. Confidence

*Use the following probes when encouraging the participants to talk and discuss:*

- Please describe!
- Does anyone have own experience or experiences you have heard of, that you want to share regarding …
- Can you explain why you think this happens in the Bangladeshi setting?
- Can you please describe how?
- Do you agree/disagree?
- Can anyone else add something?

1. ***What are the existing barriers to providing quality midwifery care in Bangladesh from a professional perspective?***
   - Apart from what we already have discussed, can you describe or discuss any other barrier from a professional perspective, which prevents quality midwifery care, as you see it?

*Let everyone tell their feeling and after that:*

Please, describe and discuss why you feel in this way

*If negative feelings, probe:*

Please, discuss what actions could be done which could change these feelings and by whom

**Closing:** Before we close up this focus group discussion, is there anything more you would like to share?

*Thank you very much for your participation!*

The recording is being turned off.

**The End**
